# Comparing perceptual judgments in large multimodal models and humans

**DOI:** 10.3758/s13428-025-02728-w

**Published:** 2025-06-19

**Authors:** Billy Dickson, Sahaj Singh Maini, Craig Sanders, Robert Nosofsky, Zoran Tiganj

**Affiliations:** 1 Department of Computer Science, Luddy School of Informatics, Computing, and Engineering, Indiana University Bloomington, 700 N Woodlawn Ave, Bloomington, IN 47408 USA; 2 Department of Psychological and Brain Sciences, Indiana University Bloomington, Bloomington, IN USA

**Keywords:** Large multimodal models, Vision-language models, Large language models, Perceptual judgments, High-dimensional similarity spaces

## Abstract

Cognitive scientists commonly collect participants' judgments regarding perceptual characteristics of stimuli to develop and evaluate models of attention, memory, learning, and decision-making. For instance, to model human responses in tasks of category learning and item recognition, researchers often collect perceptual judgments of images in order to embed the images in multidimensional feature spaces. This process is time-consuming and costly. Recent advancements in large multimodal models (LMMs) provide a potential alternative because such models can respond to prompts that include both text and images and could potentially replace human participants. To test whether the available LMMs can indeed be useful for this purpose, we evaluated their judgments on a dataset consisting of rock images that has been widely used by cognitive scientists. The dataset includes human perceptual judgments along 10 dimensions considered important for classifying rock images. Among the LMMs that we investigated, GPT-4o exhibited the strongest positive correlation with human responses and demonstrated promising alignment with the mean ratings from human participants, particularly for elementary dimensions such as lightness, chromaticity, shininess, and fine/coarse grain texture. However, its correlations with human ratings were lower for more abstract and rock-specific emergent dimensions such as organization and pegmatitic structure. Although there is room for further improvement, the model already appears to be approaching the level of consensus observed across human groups for the perceptual features examined here. Our study provides a benchmark for evaluating future LMMs on human perceptual judgment data.

## Introduction

Among the fundamental goals in the computational modeling of cognitive processes such as attention, categorization, old–new recognition, and decision-making is to account for human performance at the level of individual items. With respect to this goal, a major current trend in the psychological and cognitive sciences has involved the scaling up of the models from applications in simple, highly controlled, low-dimensional stimulus domains to complex, real-world, high-dimensional ones (Bainbridge, 2019; Battleday et al., 2020; Hebart et al., 2020; Hout & Goldinger, 2015; Meagher & Nosofsky, 2023; Nosofsky, Meagher, & Kumar, 2022; Nosofsky et al., 2018a, 2018b; Storms et al., 2000). Another current trend is to scale up the application of the models to cases in which performance is modeled for very large numbers of individual items from the domains of interest (Battleday et al., 2020; Hebart et al., 2020; Kramer et al., 2023; Meagher & Nosofsky, 2023).

Applying the models for predicting individual-item performance requires the specification of a psychological “feature space” in which the items are embedded. Historically, one of the major approaches to deriving such an input space for cognitive models has been to conduct independent tasks involving the collection of varied forms of “similarity-scaling” data (Nosofsky, 1992; Roads & Love, 2024). For example, observers might be required to make direct judgments of the similarity between different pairs of items (Shepard, 1962); judge whether pairs of presented items are the “same” or “different” (Kruskal & Wish, 1978; Rothkopf, 1957); choose which item among a triad of presented items is the “odd one out” (Hebart et al., 2020; Roads & Mozer, 2019); or form spatial arrangements of entire sets of objects in which the distance between objects is proportional to their judged dissimilarity (Goldstone, 1994; Hout, Goldinger, & Ferguson, 2013). Formal statistical models can then be applied for modeling the obtained similarity-scaling data by embedding the judged items in derived multidimensional feature spaces (Hout, Papesh, & Goldinger, 2012; Lee, 2001; Shepard, 1962, 1980, 1987)

In modern work, however, a limitation of this classic approach is that the techniques eventually become intractable when the number of to-be-scaled items is extremely large and/or when the dimensionality of the feature space is large and complex (but for modern efforts along these lines, see Hebart et al., 2020; Marjieh et al., 2024; Nosofsky et al., 2018a, 2018b). For example, to fill out a similarity-judgment matrix with just a single judgment for all pairs of *n* items requires something on the order of *n*^2^ similarity judgments. If *n* = 1,000, then something on the order of 1,000,000 judgments are needed.

Therefore, as an alternative to this similarity-scaling approach, a major current trend is for researchers to use a variety of machine-based deep-learning network approaches for deriving the feature space to be used as input to the cognitive-process models (Annis et al., 2021; Battleday et al., 2020; Lake et al., 2015; Peterson et al., 2018; Roads & Love, 2021). The general approach here is to train deep-learning models to learn to classify large sets of items into different categories in a domain of interest. By using appropriate validation and generalization techniques to avoid overfitting the noise in the data, the respective models learn a set of weights that can classify with high accuracy both the trained items and novel items from the relevant domains. Once the training has been completed, the deep-learning model can then be used as a mechanism for embedding the items in a candidate feature space. In particular, the presentation of any given item will give rise to a set of activations of the nodes in the network. Oftentimes, transformations of the node activations at the penultimate layer of the network, immediately prior to the final classification layer, are chosen as the candidate features. These candidate features are then used as inputs to a variety of cognitive-process models used for predicting human performance in related tasks and domains.

Although some impressive successes have been achieved with these deep-learning approaches, they also have potential limitations. One limitation is the lack of assurance that all stimulus features detected by the machine-learning models are similarly apprehended by humans. To take a hypothetical example, suppose that a particular machine-learning model can apprehend wavelengths of light or sound that go beyond human sensitivities and that these “extrasensory” features turn out to be highly diagnostic for purposes of classification. In that case, the candidate feature space derived from the deep-learning model would include nonhuman features, so the use of that feature space in combination with cognitive models could lead to misleading conclusions involving the nature of human performance. Indeed, there is evidence that machine-learning models make use of features that are not meaningful to human observers. It is well known that images can be subtly modified to drastically alter the predictions of computer vision models (e.g., causing models to classify a bus as an ostrich), even if the altered and unaltered images look identical to the human eye (Szegedy, 2013).

A related issue is that oftentimes it is extremely difficult to place a psychological interpretation on the patterns of penultimate-layer activations. By comparison, in our view, the psychological interpretations of the dimensions that are generally achieved via similarity-scaling techniques can yield deeper insights into the nature of people’s mental representations of the objects. Still another limitation is that there is an enormous set of highly complex nonlinear transformations that take place in translating elementary input features in the networks into the patterns of activations at the penultimate layers. Thus, the machine-learning-based feature space that is used as an input to the cognitive models may not correspond to the types of foundational psychological building-block features that are supposed by the cognitive models in the first place.

To address these limitations, Sanders and Nosofsky (2018, 2020) proposed a hybrid two-step approach (see also Rumelhart & Todd, 1993; Steyvers & Busey, 2000). The approach was intended to combine the strengths of the similarity-scaling and deep-learning approaches to deriving psychological feature spaces for large numbers of real-world high-dimensional objects. In the first step, one uses traditional similarity-scaling methods for deriving a feature-space representation for a representative *subset* of the objects in the domain of interest. In the second step, rather than training a deep-learning network to classify objects in this domain into categories, one instead trains the deep-learning network to reproduce the actual feature-space representation for the subset of objects that was derived from the *human* judgments. Again, by using appropriate validation and generalization techniques to avoid overfitting the training-item representation, the approach could allow for the embedding of an essentially infinite number of objects from the domain of interest in a high-dimensional *psychological* feature space.

A further advantage of the proposed two-step approach is that the same technique can be applied for positioning objects along additional dimensions not revealed by the similarity-scaling techniques themselves. For example, Sanders and Nosofsky (2020) tested the proposed approach in a domain involving igneous rock categories as defined in the geological sciences. Using similarity-scaling techniques, Nosofsky et al., (2018a, 2018b) had previously found that an eight-dimensional multidimensional scaling (MDS) representation provided an excellent account of similarity-judgment data obtained for a set of 360 rock images. In addition, the derived MDS dimensions had natural psychological interpretations. These included the extent to which the rocks were (i) light or dark, (ii) fine- or coarse-grained, (iii) smooth or rough, and (iv) dull or shiny, (v) had disorganized versus organized textures, (vi) were achromatic versus chromatic, and (vii) had a green versus red hue. (A firm interpretation was not obtained for the eighth dimension, but it appeared to have shape-related components.) Crucially, however, Nosofsky et al. (2020) and Sanders and Nosofsky (2020) discovered that in independently conducted classification-learning experiments, observers made use of additional “supplementary” dimensions that were not revealed by the initial MDS analysis but that were highly diagnostic for purposes of classifying the rock images into their geologically defined categories. One example included the extent to which the rocks possessed “porphyritic texture”—the embedding of small fragments within a fine-grained groundmass. (We describe other examples of these supplementary dimensions below.) In previous research projects, Nosofsky et al., (2018a, 2018b, 2020) obtained direct ratings from human subjects of the positions of all the rock images along each of the MDS-derived and supplementary dimensions. Importantly, the two-step approach proposed by Sanders and Nosofsky (2020) for training deep-learning networks to reproduce MDS-derived representations also showed some preliminary success in reproducing the position of the rocks along these supplementary dimensions.

Despite the initial promise of the proposed two-step method, Sanders and Nosofsky (2020) acknowledged that improved machine-learning technologies would likely yield more effective procedures for implementing it. Indeed, in the short time since the original proposal, an explosion of such improved technologies has emerged.

In recent years, multimodal vision and language models have demonstrated the ability to produce advanced image understanding. These models combine inputs from both visual and textual sources to understand and generate content that reflects a combined understanding of both modalities. Multimodal vision and language architectures such as CLIP (Radford et al., 18–24 Jul 2021) demonstrate powerful zero-shot learning, surpassing humans in zero-shot, one-shot, and two-shot learning on the Oxford IIT Pets image classification dataset. When embedded into larger frameworks such as OpenAI’s GPT-4 (OpenAI et al., 2023) or GPT-4o (OpenAI et al., 2024), Google’s Gemini (Gemini Team et al., 2024), or Anthropic’s Claude-3 (Anthropic, 2024), which all have multiple training components, including reinforcement learning from human feedback (Glaese et al., 2022), these multimodal models gain additional power and flexibility in terms of interaction with the human users. Careful design of prompts, specifically techniques such as chain-of-thought prompting, can further increase the performance of these models (Lin, 2024; Wei et al., 2022). Kouwenhoven et al. (2022) highlight the importance of developing shared vocabularies between humans and machines, proposing that natural evolution in communication could significantly improve AI interactions. Recent studies investigated relationships between humans and models more systematically by quantifying differences across specific perceptual features (Geirhos et al., 2021; Sheybani et al., 2024) and visual illusions (Shahgir et al., 2024), complemented by an overview by de Kleijn (2022) outlining the evolution of AI and neural networks in relation to the human brain.

The central purpose of the present work was to investigate the effectiveness of the current technology using large multimodal models (LMMs) for positioning large sets of complex, real-world objects in high-dimensional feature spaces that align with human judgments. Following Sanders and Nosofsky (2020), our example target domain involves the set of 360 rock images used in their earlier study. This domain is particularly suitable for our current investigation due to the substantial amount of previously collected psychological-scaling and direct dimension-rating data for these rock images. Consequently, the predictions generated by LMMs can be rigorously compared to the extensive existing sets of human judgments.

In the present work, we conduct an experimental investigation of the performance of LMMs that accept image and text inputs, specifically OpenAI GPT-4 and GPT-4o and the Anthropic Claude-3 model family, for reproducing human dimension ratings within the rock domain. Our investigation is structured around two primary implementation conditions. In each condition, we evaluate the correspondence between the ratings produced by LMMs and human ratings along a set of seven MDS-derived dimensions and three supplementary dimensions. In the first condition, for each to-be-judged image, we provide LMMs with verbal prompts that are identical to the verbal prompts that were provided to human subjects in the previous dimension-rating studies. This first condition has certain similarities to an important investigation recently reported by Marjieh et al. (2023). Using only verbal prompts, these researchers asked GPT-4 and some related machine-learning systems to make judgments of similarity for stimuli in six domains, such as tones varying in pitch or colors varying in wavelength. They then obtained MDS solutions for the GPT-4-produced similarity ratings and found that the recovered MDS solutions corresponded well with ones derived in past studies in which humans provided similarity judgments of the actual perceptual stimuli. By contrast, in Condition 1 of the present study, on each trial we present to the machine-learning system an actual rock image to be judged. We then use verbal prompts to ask the machine-learning algorithm to produce its dimension ratings for the image. The system is asked to produce direct ratings along 10 different dimensions that are components of a single set of complex rock-image stimuli.

In the second condition of our investigation, we supplement the verbal prompts with a set of images that illustrate examples with low, medium, and high values along the rated dimensions. These “anchor images” were in fact used in the studies conducted by Nosofsky et al., (2018a, 2018b, 2020) in which the human dimension ratings for the rocks were originally collected. We hypothesized that the correspondence between the multimodal machine-learning ratings and the human ratings would be significantly improved when the system was provided with the example anchor images. Finally, as we explain in our general discussion, we followed up our primary investigations in these two main conditions with some additional exploratory investigations that varied the detailed nature of the prompts as well as different methods of aggregating the perceptual-rating predictions of the LMMs.

## Methods

We used previously collected data from human participants who performed dimension ratings of 360 rock images across a set of continuous and present–absent dimensions (Meagher & Nosofsky, 2023; Nosofsky et al., 2018a, 2018b). The complete dataset, including rock images and participant ratings, is publicly available through OSF: https://osf.io/cvwu9/.

### Stimuli

The stimuli were 360 images of rocks obtained from the web and processed to remove background objects and idiosyncratic markings such as text labels. The 360 images included 120 rocks from each of three main divisions: igneous, metamorphic, and sedimentary. Each main division included 12 rocks from 10 major subtype categories (30 subtypes in total), such as granite, marble, sandstone, and so forth.

For human experiments, the stimuli were presented on a 23-in. LCD computer screen. The stimuli were displayed on a white background. Each rock picture was approximately 2.1 in. wide and 1.7 in. tall. Subjects sat approximately 20 in. from the computer screen, so each rock picture subtended a visual angle of approximately 6.0° × 4.9°. Images were selected or digitally manipulated to have similar levels of resolution of the salient features that may be used to identify and classify the particular rock types. All the images were photographed in a field setting and had not been modified in any way other than the removal of other portions of the original image.

### Human dimension ratings

After excluding dimensions that did not have complete participant data, we focused our analysis on 10 dimensions, seven derived from MDS analysis of similarity judgments (chromaticity, darkness/lightness, disorganized/organized, dull/shiny, fine/coarse grain, red/green, and smooth/rough) and three “supplementary” dimensions (conchoidal fracture, pegmatitic structure, and porphyritic texture) that were not revealed by the MDS analysis but that were added due to being highly diagnostic for purposes of classifying the rock images into their geologically defined categories.^1^ Each of these dimensions had a continuous rating scale from 1 to 9.

### Multimodal vision and language models

We employed state-of-the-art LMMs GPT-4, GPT-4o, and the Claude-3 model family. The Claude-3 model family includes the three models Opus, Sonnet, and Haiku, with Opus reported to be the most intelligent model and Haiku being the least intelligent but the fastest model amongst the three models (Anthropic, 2024). All the models used in the experiment were prompted using API calls. In order to minimize randomness in the responses, the temperature parameter was set to 0 for all the models. Prompts to the models were identical to those provided to human subjects aside from minor modifications instructing the model to return a numerical dimension rating to the hundredths place^2^ and changing plural references to describe a single trial, as each API call to the model was independent. Furthermore, in the condition without anchor images (Condition 1), any text mentioning the presence of example images was omitted from the prompt. RGB images were used to prompt the models in Condition 2, in which anchor images were part of the prompt. All prompts are listed as part of the Supplementary Information available at https://osf.io/za847/.

**Fig. 1:**
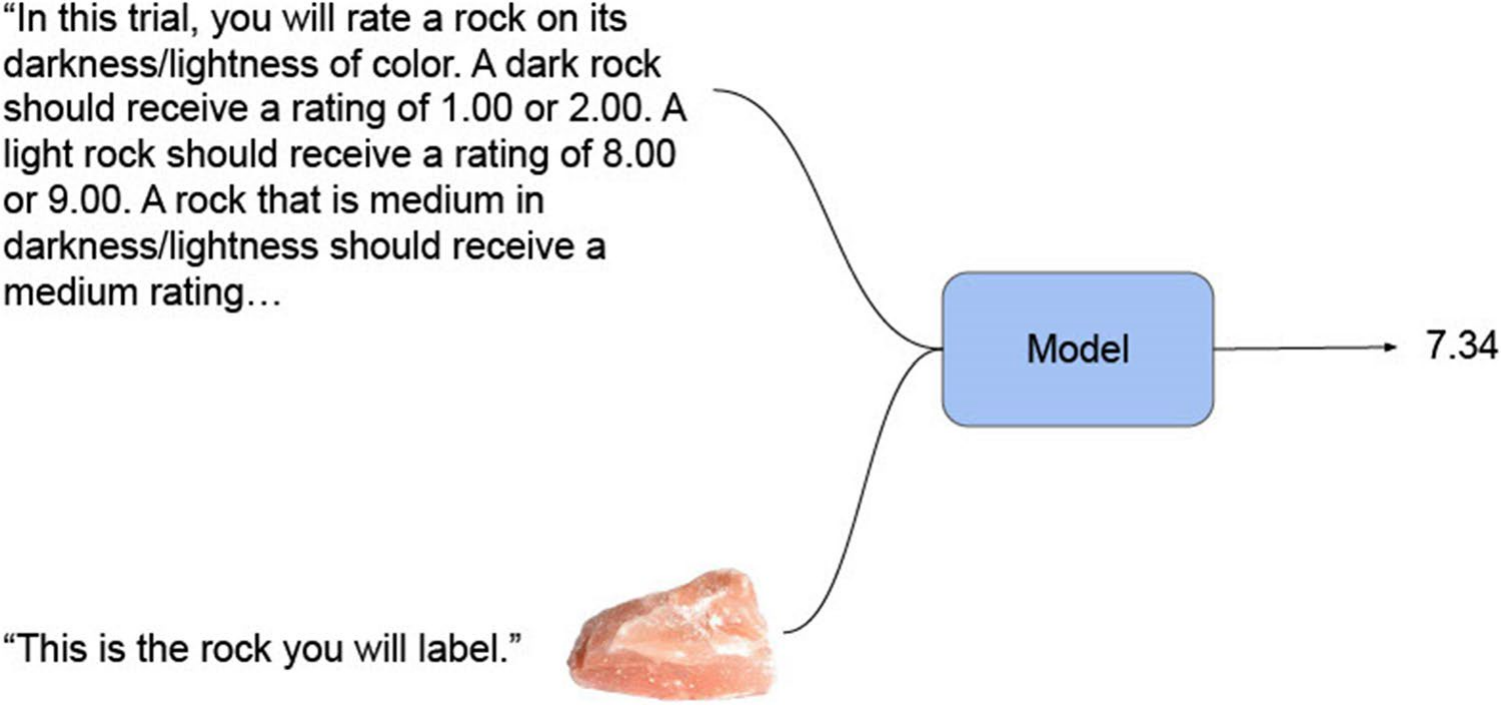
Prompting pipeline for Condition 1

In the first condition, 360 images were provided to each of the five models for each of the 10 different dimensions. No anchor images were provided to the model. For GPT-4o and GPT-4 specifically, the text “Do not respond with I’m sorry…” was added to the prompt of several features (fine/coarse grain, red/green hue, porphyritic texture, pegmatitic structure, and conchoidal fracture) in order to minimize redundant responses from the model. An example of this condition is displayed in Fig. 1.

In this example, the prompt for darkness/lightness is passed to the model along with the rock image to be rated on a scale of 1 to 9. The model produces one numeric value.

**Fig. 2:**
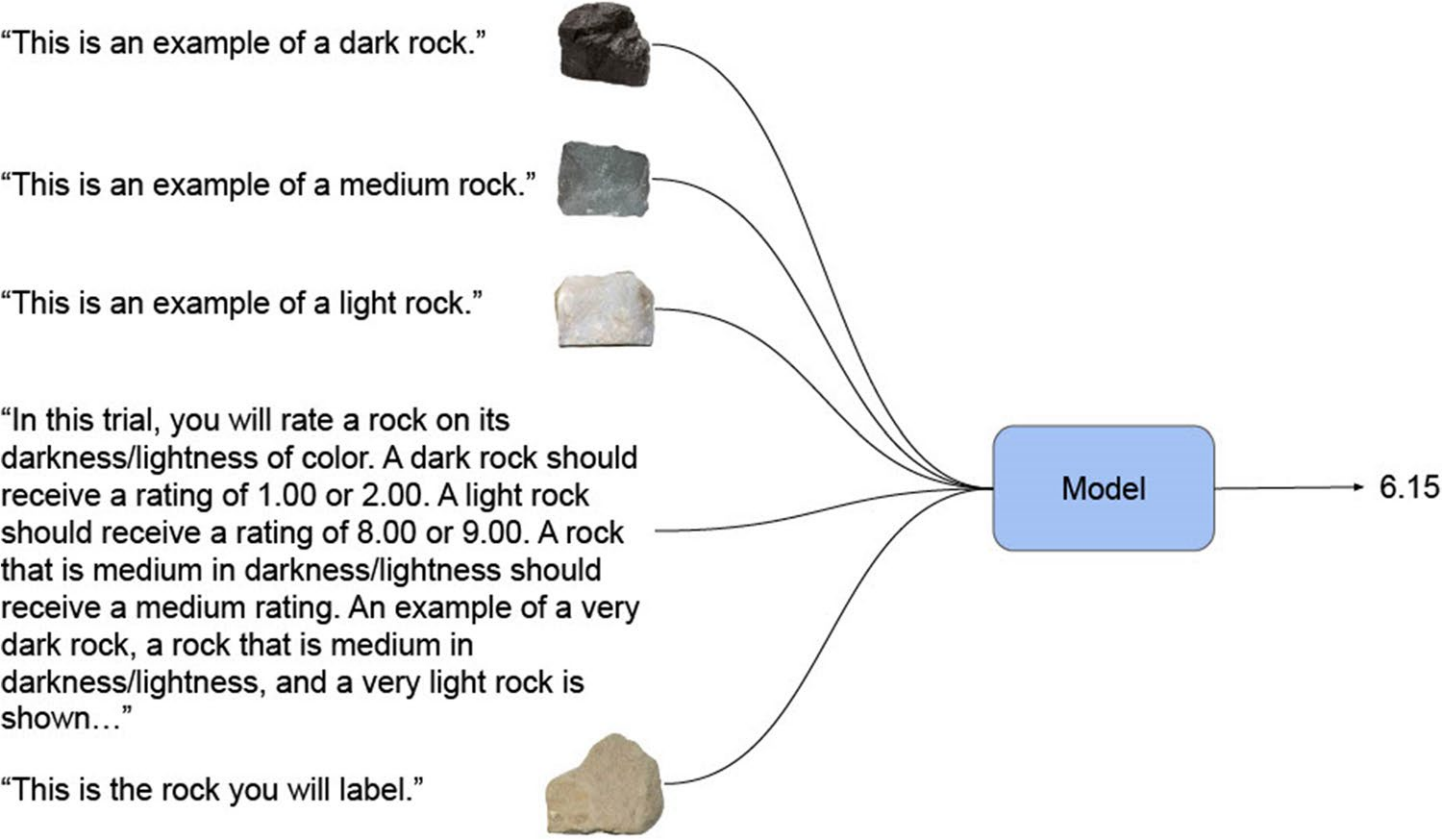
Prompting pipeline for Condition 2

In the second condition, we added three anchor images to each prompt. The anchor images were combined with verbal indicators for the feature being tested (e.g., “This is an example of a dark rock”). Anchor images included examples with low, medium, and high values along the rated dimensions. We used the same anchor images that were presented to the participants in the previous human dimension-rating studies from Nosofsky et al., (2018a, 2018b, 2020). An example of this condition with anchor images is provided in Fig. 2.

In this example, three anchor images depicting dark, medium, and light rocks are passed to the model with the darkness/lightness feature prompt and the rock to be rated. The model again produces one numeric value.

## Results

### Preliminary summary of the main pattern of results

Before providing a detailed report of comparisons among models across the conditions, we start by presenting the results from GPT-4o in Condition 1. Across both conditions, GPT-4o was the best-performing model overall. In addition, as will be seen, adding anchor images led to relatively small changes in GPT-4o’s performance relative to the purely verbal prompts used in Condition 1. The initial report that we provide in this section is intended to give the reader an immediate sense of both the strengths and weaknesses of these multimodal systems in reproducing human judgments along these varied dimensions of the rock images.

**Fig. 3:**
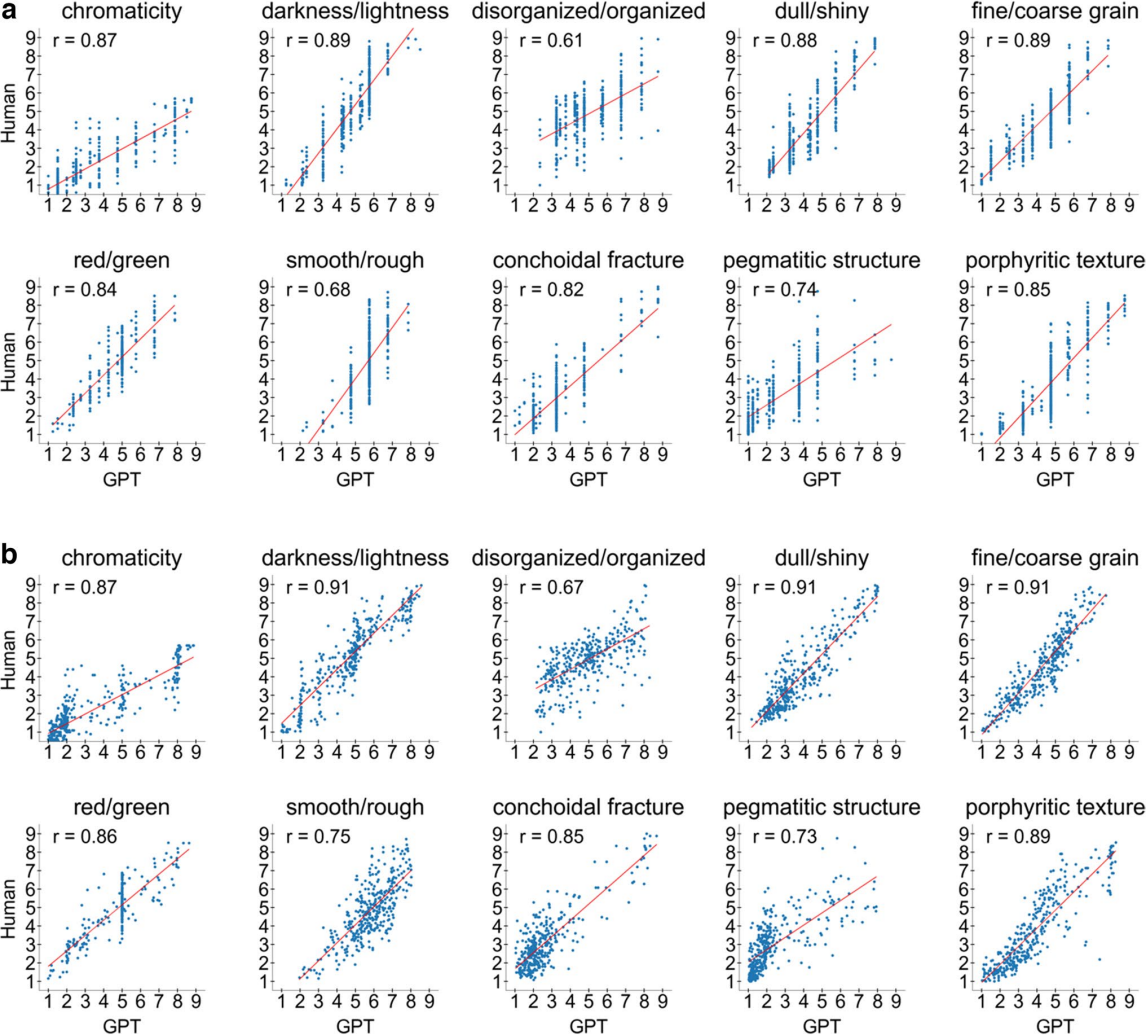
** a** Scatterplots of human and GPT-4o ratings in Condition 1 (without anchor images). The Pearson correlation coefficients are shown in the upper left, and the regression lines are shown in red. **b** Scatterplots of human and GPT-4o-aggregate ratings in Condition 1 (without anchor images). The Pearson correlation coefficients are shown in the upper left, and the regression lines are depicted in red. Details on the aggregation method can be found in the "Exploratory investigations and future research directions" section of the discussion

We display in Fig. 3a scatterplots of GPT-4o’s Condition-1 results for each of the 10 dimensions. Each panel plots the mean observed human ratings for the 360 rock images against the ratings produced by GPT-4o. The correlation between the observed and predicted ratings is also reported. As explained later in our article, we have also constructed interactive versions of each scatterplot with more detailed information than shown here. The interactive scatterplots are available at https://cognlp.com.

As can be seen in Fig. 3a, there is some variation in the quality of GPT-4o’s predictions across the different dimensions, with the correlations ranging from *r* = 0.61 for disorganized/organized to *r* = 0.89 for darkness/lightness. In general, GPT-4o performs best in matching the human ratings for what seem to us to be relatively “elementary” visual dimensions such as darkness/lightness, chromaticity, dull/shiny, and fine/coarse grain texture. The correlations for these elementary dimensions are reasonably high, and the scatterplots show that there is appropriate variation across the entire range of ratings. (In other words, the high correlations are not the result of the model predicting only very low ratings for one subset of stimuli and only very high ratings for a second set.) However, GPT-4o’s performance declines for what seem to us to be more abstract and emergent dimensions such as organization and pegmatitic structure. In our general discussion, we consider other possible reasons for the variation in GPT-4o’s performance across the different dimensions. Interestingly, we will see that humans also show less agreement among themselves in their ratings of the more abstract dimensions compared to the elementary ones.

In testing these multimodal systems, our hope was that their ratings might show sufficient reliability and correspondence with the mean human ratings to make it unnecessary to collect extensive human ratings in future research. As we will see in our detailed report below, GPT-4o showed significant promise, achieving a level of reliability comparable to individual human raters and even surpassing this level for certain dimensions, particularly for elementary ones. However, we will see that there remains room for improvement, as its correlations still fall short of the level of reliability in the mean human group ratings. While the best-performing systems provide dimension ratings that correlate with the mean human ratings at levels as good as or better than those of individual participants, the suitability of this degree of reliability will depend on the specific objectives of the research.

## Detailed evaluation of the LMMs across the two primary conditions

We first evaluated each of the five models: GPT-4o (OpenAI et al., 2024), GPT-4 (OpenAI et al., 2023), and Haiku, Sonnet, and Opus (three variants of the Claude-3 model from Anthropic, 2024). The results of the evaluation on Condition 1 (which included only verbal prompts) and Condition 2 (which included anchor images) are shown in Table 1.

**Table 1:** Correlations between multimodal model ratings and averaged human ratings in Conditions 1 and 2 (with and without anchor images). For each dimension, the highest correlation among the five models in both conditions is indicated in boldface font

dimension/model	GPT-4o	GPT-4o with anchors	GPT-4	GPT-4 with anchors	Haiku	Haiku with anchors	Sonnet	Sonnet with anchors	Opus	Opus with anchors
Chromaticity	**0.87**	0.85	0.77	0.80	0.81	0.82	0.80	0.77	0.72	0.80
Darkness/lightness	**0.89**	**0.89**	0.88	0.85	0.86	0.87	0.84	**0.89**	0.79	0.83
Disorganized/organized	0.61	**0.62**	0.48	0.42	0.25	0.12	0.45	0.57	0.28	0.33
Dull/shiny	**0.88**	0.87	0.81	0.72	0.40	0.48	0.75	0.61	0.33	0.47
Fine/coarse grain	0.89	**0.90**	0.76	0.80	−0.02	0.53	0.61	0.76	0.25	0.46
Red/green	**0.84**	0.83	0.82	0.78	0.53	0.71	0.67	0.83	0.59	0.82
Smooth/rough	0.68	**0.73**	0.48	0.63	0.04	0.24	0.32	0.68	0.19	0.42
Conchoidal fracture	**0.82**	0.81	0.67	0.71	0.30	0.53	0.55	0.70	0.29	0.43
Pegmatitic structure	**0.74**	**0.74**	0.43	0.56	0.28	0.32	0.38	0.66	0.20	0.57
Porphyritic texture	**0.85**	**0.85**	0.66	0.67	0.59	0.43	0.59	0.65	0.58	0.55

Overall, GPT-4o outperformed all other models across every dimension, except for the darkness/lightness dimension, where it matched Sonnet with anchors. Within the Claude-3 family, the cheapest model to use, Haiku, performed best on chromaticity, and the medium-priced model, Sonnet, outperformed Haiku and Opus on the remaining nine dimensions. Adding anchor images yielded mixed performance improvements across models. For GPT-4o, performance remained basically the same. For Haiku, performance increased in eight of ten features, with decreases in disorganized/organized and porphyritic texture. For Sonnet, performance improved across all dimensions except chromaticity and dull/shiny. For Opus, performance increased across all dimensions except porphyritic texture.

**Fig. 4:**
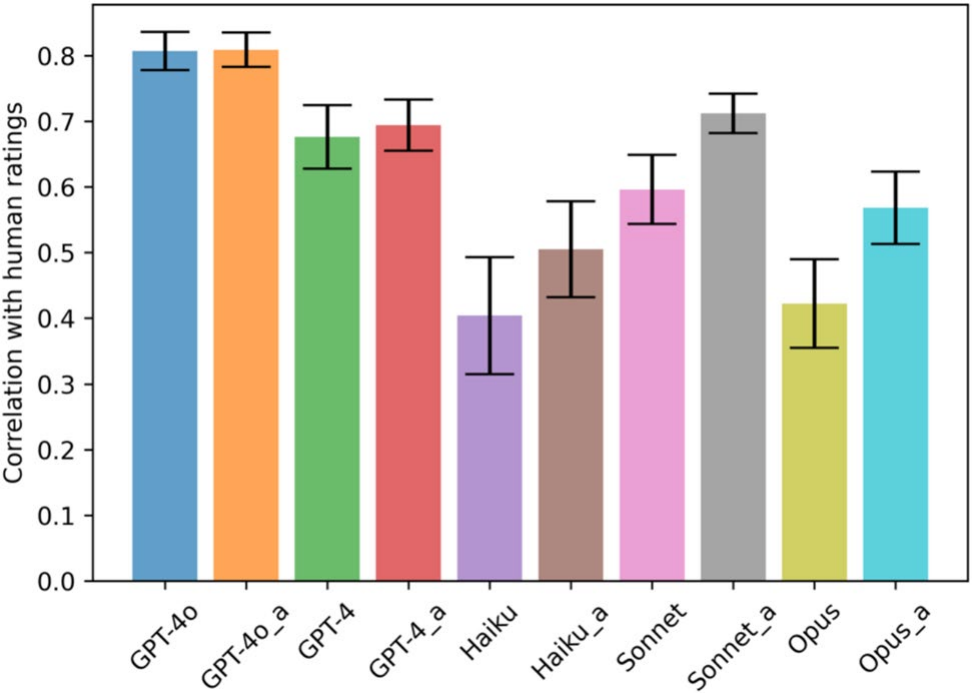
Mean and standard deviation of correlation between the mean rock ratings of human subjects and LMMs in Conditions 1 and 2 (“a” refers to prompts with anchor images)

Figure 4 shows, for each multimodal model in Conditions 1 and 2, the mean correlation across all the rock dimensions between the ratings of the human subjects and the models. Adding anchor images increases the mean correlation and decreases the standard deviation of the correlations across all models. However, the overall improvement yielded by the use of the anchors in the case of GPT-4 and GPT-4o is rather small.

### Variance in human perceptual judgment data

To better understand the performance of the LMMs in relation to the ratings data obtained from human participants, we conducted two analyses to quantify the variance in the human perceptual judgment data. In the first analysis, for each dimension, we computed the correlation coefficient of the 360 ratings between each of the 20 individual subjects and the mean rating produced by the remaining 19 subjects. For example, we computed the correlation between subject 1’s ratings and the mean ratings of subjects 2–20; the correlation between subject 2’s ratings and the mean ratings of subjects 1 and 3–20; and so forth. We then computed the mean and standard deviation of each of these 20 individual-subject/mean-rating correlations. The results are given in the first column of Table 2. For ease of comparison, in columns 3 and 4, we also again present the best-performing runs for GPT-4o in Conditions 1 and 2. In general, the model’s correlations with the mean human ratings are roughly the same as or sometimes substantially higher than the correlations of the individual subjects’ ratings with the mean ratings.

**Table 2:** Correlation in human ratings (first two columns) and between human ratings and best-performing LMM runs (columns 3–8)

Dimension	Mean individual- subject rating correlations ± *SD*	Mean split-half group-rating correlations ± *SD*	GPT-4o Cond. 1	GPT-4o Cond. 2	Average Cond. 1	Average Cond. 2	GPT-4o Aggregate Cond. 1	GPT-4o Aggregate Cond. 2
Chromaticity	0.79 ± 0.07	0.94 ± 0.01	0.87	0.85	0.87	0.87	0.87	0.86
Darkness/lightness	0.87 ± 0.05	0.97 ± 0.00	0.89	0.89	0.93	0.92	0.91	0.91
Disorganized/organized	0.66 ± 0.09	0.89 ± 0.02	0.61	0.62	0.61	0.56	0.67	0.67
Dull/shiny	0.80 ± 0.06	0.95 ± 0.01	0.88	0.87	0.83	0.88	0.91	0.89
Fine/coarse grain	0.76 ± 0.09	0.94 ± 0.01	0.89	0.90	0.90	0.83	0.91	0.91
Red/green	0.85 ± 0.03	0.97 ± 0.00	0.84	0.83	0.90	0.87	0.86	0.85
Smooth/rough	0.74 ± 0.07	0.93 ± 0.01	0.68	0.73	0.76	0.53	0.75	0.80
Conchoidal fracture	0.68 ± 0.10	0.86 ± 0.02	0.82	0.81	0.85	0.78	0.85	0.83
Pegmatitic structure	0.67 ± 0.07	0.90 ± 0.02	0.74	0.74	0.77	0.59	0.73	0.77
Porphyritic texture	0.79 ± 0.08	0.95 ± 0.01	0.85	0.85	0.82	0.78	0.89	0.85

In a second analysis, we computed split-half correlations for each dimension to quantify reliability at the level of the mean ratings themselves. In this analysis, we conducted 1,000 simulations for each dimension. For each simulation, we divided the 20 subjects who gave ratings into two equally sized random groups. We then computed the mean rating of each of the 360 rocks on that dimension for each group, and finally computed the correlation between the 360 ratings across the two groups. In column 2 of Table 2, we report the mean and standard deviation (across the 1,000 simulations) of these split-half correlations for each of the dimensions. As can be seen, these split-half correlations are consistently higher than those produced by GPT-4o. Nevertheless, although there is still room for improvement, in our judgment, many of the correlations achieved by GPT-4o are quite impressive, and the system might already be suitable for providing adequate perceptual-dimension ratings for a variety of research purposes.

Finally, in the remaining columns of Table 2 (columns 5–8), we report results from a variety of exploratory investigations involving model-aggregation schemes that we describe in our general discussion.

### Interactive visualization for individual rock scores

**Fig. 5:**
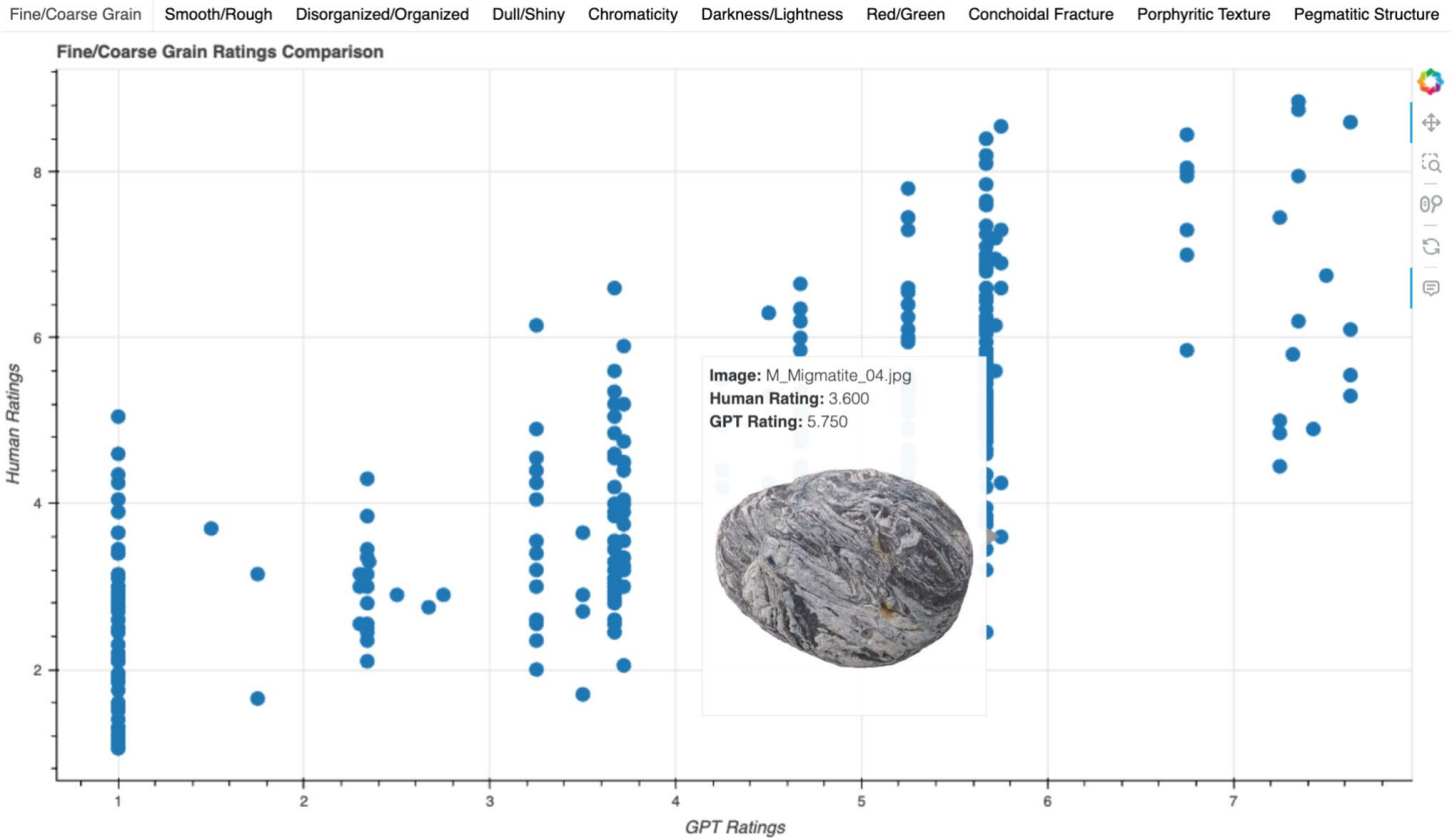
Example of an interactive plot. Users select a plot for a specific condition and then select a specific feature dimension to view the correlation plot. When a specific datapoint is hovered over, rocks with that combination of scores appear, displaying the name of the specific rock image and the human and model rating for that rock

For individual rock image results, we have developed an interactive visualization available at cognlp.com where users can view correlation plots for each feature and each condition and hover over each data point to view the specific human and model ratings for that rock. Figure 5 shows an example of the interactive plots. These interactive visualizations are useful, for example, for identifying specific mispredictions from the models that could help guide future modifications and improvements.

## General discussion

### Summary

Recent rapid advancements in large multimodal neural networks have led to the development of models that can respond to verbal prompts about image properties, demonstrating zero-shot learning. These models can also take images as part of the input prompt, enabling one-shot and few-shot learning. It has been demonstrated that model performance can exceed human performance at many tasks, such as image classification, image captioning, and visual question answering (He et al., 2015; Li et al., 2023; Wang et al., 2022a, 2022b; Weihan Wang et al., 2023; Wenhai Wang et al., 2022a, 2022b; Yang et al., 2022). Here, we investigated whether the models are capable of mimicking humans in providing ratings of visual perceptual dimensions composing complex objects. To the extent that the LMMs are successful at this task, collecting the LMM ratings could provide a promising approach to embedding large numbers of complex objects in high-dimensional feature spaces defined along human psychological dimensions.

We compared the performance of multimodal vision and language models to human performance in a perceptual judgment task. We used an existing dataset where human participants were asked to make dimension ratings for a series of rock images along different dimensions that were either found through MDS scaling of human similarity judgment data or deemed important for classifying the rock images into their geologically defined categories. This dataset has been extremely valuable in cognitive science for investigating human category learning and evaluating machine-learning classification (Cerone et al., 2022; Lu, Penney, & Kang, 2021; Mandal, Deborah, Tobing et al., 2024; Meagher & Nosofsky, 2023; Nosofsky et al., 2020, 2022; Nosofsky et al., 2018a, 2018b). Its application with LMMs presented here constitutes a novel approach to evaluating how close the models are to human performance in terms of judging perceptual features.

Our work provides a starting tool that can be used by the research community to quantify the performance of multimodal vision and language models in communicating with human users about perceptual dimensions. Our finding that GPT-4o provided clearly better results than GPT-4, Sonnet, Haiku, and Opus provides an example of the utility of this benchmark score. We tested all the models across two main conditions. In the first, we provided the LMMs with purely verbal prompts describing the to-be-rated dimensions along with the to-be-rated images; in the second, we supplemented the verbal prompts with example anchor images for illustrating the to-be-rated dimensions. Although the use of the anchor images led to improved performance for Sonnet, Haiku, and Opus, their performance still fell far short of the results achieved by GPT-4o. The use of the example anchor images led to only minor overall improvements for GPT-4o.

A specific purpose of the present investigation was to shed light on the possibility of replacing human participants' perceptual judgment collection with ratings provided by LMMs. This line of work has recently attracted a significant amount of attention (Aher et al., 2022; Argyle et al., 2023; Demszky et al., 2023; Dillion et al., 2023; Marjieh et al., 2023). Our study is novel in that it focuses on perceptual judgments of LMMs of a specific psychological dataset composed of visual stimuli and human ratings along a large set of domain-relevant perceptual dimensions. On the one hand, our results indicate that the correlations between GTP-4o’s ratings and the mean human ratings were as high or higher than the correlations between individual subjects’ ratings and the mean human ratings. Moreover, for “elementary” dimensions such as darkness/lightness and chromaticity, the correlations between GPT-4o’s ratings and the mean human ratings were quite high in an absolute sense, approaching values of *r* = 0.90. For certain research purposes, this impressive level of performance may already provide a useful starting point, such as for curating stimulus sets for use in pilot studies. On the other hand, GPT-4o’s correlations with the mean human ratings were lower for more abstract and emergent dimensions, such as organization (*r* = 0.62) and pegmatitic structure (*r* = 0.74). Furthermore, for all dimensions, GPT-4o’s correlations always fell short of the split-half correlations associated with the mean human ratings. Thus, although our reported results are extremely promising, there is still room for improving the LMMs’ abilities to reproduce human perceptual judgments.

An interesting question is why the LMMs’ correlations with the mean human judgments tended to be lower for the more abstract dimensions than for the elementary ones. In answering this question, we should first note that the correlations of individual subject ratings with mean ratings also tended to be lower for the more abstract dimensions (see Table 2). One possibility is that, whereas elementary dimensions like lightness or chromaticity might be consistent across numerous domains, making it easier for both humans and models to learn and transfer knowledge, more abstract dimensions might be specific to certain contexts or domains. For example, the pegmatitic structure of a rock might not have a direct analog in other domains that humans and models have been exposed to, leading to more variable performance in those specific dimensions.

### Exploratory investigations and future research directions

A goal of future research is to improve upon the promising results already achieved in the present research. We have initiated a variety of new investigations that are exploratory in character but not highly systematic. We briefly describe these exploratory analyses and future directions in the present section.

In one set of investigations, we focused on whether the models’ predictions for the abstract dimensions of organization and pegmatitic structure could be improved through the use of modified prompts. We used the interactive scatterplots illustrated in Fig. 5 to identify specific outliers in which the models’ predictions departed severely from the human data. Using the outliers as a guide, we then attempted to develop more extended verbal prompts that we thought might remedy the models’ limitations for these dimensions. For example, whereas humans seemed to be relying on judgments of “global” organization in making ratings on this dimension, it appeared that GPT-4 gave high ratings in cases in which local components of the image were highly organized. Our modified prompt therefore attempted to emphasize the importance of “global organization” in the definition of this dimension (see Supplement for details). In addition, instead of using only three anchor images for illustrating the dimensions, we used nine anchor images that spanned the range from low to high ratings. Unfortunately, as reported in the Supplement, neither manipulation led to improved predictions.

In a second set of analyses, we investigated whether different forms of aggregation might yield improved predictions across the 10 dimensions of the rock images. In one form of aggregation, we asked whether aggregating predictions across the separate models might yield improved predictions. For example, in one such analysis, we simply averaged the predictions from the three best-performing models (GPT-4o, GPT-4, and Sonnet) for all 360 rock images across all 10 dimensions. We then computed the correlation between these model-averaged ratings and the mean human ratings. As we report in columns 5 and 6 of Table 2, although there was a slight increase in the overall correlation averaged across all 10 dimensions, whether the model-averaging procedure yielded improvements or decrements varied across the individual dimensions.

In a second form of aggregation, we followed the lead of Sanders (2024), who reported results of a study conducted in parallel with the present one with a subset of 30 of the 360 rock images considered in this article. Sanders (2024) generated predictions by asking GPT-4o to provide an integer rating for each individual rock along each dimension, and he asked the model to provide its top 20 responses to each prompt along with the log-probability of each response. (Asking for the “log-probability” is the specific option that is currently allowed by OpenAI’s API.) After filtering out any responses that failed to answer the prompt, he then computed a probabilistically weighted average of the ratings to yield a mean-predicted rating. This procedure may capture, at least in part, the forms of individual human-rater variability that underlie the mean human-ratings data. We applied this weighted-averaging procedure to the present data, and the results are reported in columns 7 and 8 of Table 2. As can be seen, this aggregation scheme led to small but systematic improvements in the obtained dimension correlations compared to the ones achieved when only the highest-probability rating was used to generate predictions. We display in Fig. 3b scatterplots of GPT-4o’s probabilistically weighted predictions for each of the 10 dimensions in Condition 1 (i.e., the no-anchor condition). Note that whereas the version of GPT-4o that used only the highest-probability rating led to predicted ratings that were discrete in character (Fig. 3a), the probability-weighting aggregation scheme produces ratings that are more nearly continuous. Of course, there is an extremely wide variety of different aggregation schemes that need to be systematically investigated in future research. This exploratory analysis suggests that the use of such aggregation schemes is an extremely promising future direction.

Finally, in the present article, we focused mainly on dimensions derived from MDS analyses of pairwise similarity-judgment data in evaluating the extent to which LMMs produce perceptual judgments that correlate with the judgments of humans. An alternative approach might be to ask the LMM to provide the raw similarity judgments for the pairs of images themselves. MDS could then be used to analyze the LMM-generated similarity-judgment data. Sanders (2024) reported promising results for the subset of 30 rock images used in his study, with the derived MDS dimensions corresponding reasonably well to those derived from analysis of human pairwise similarity-judgment data (Nosofsky et al., 2018a, 2018b). There is no guarantee that the LMMs will not make use of “nonhuman” dimensions in generating their similarity judgments; nevertheless, this future direction is another intriguing one that could potentially allow for an efficient method of embedding large numbers of complex objects in high-dimensional similarity spaces.

As these technologies continue to evolve, their ability to replicate human perceptual judgments across a wider range of dimensions is likely to advance, paving the way for broader applications in cognitive modeling and other domains. This advancement is likely to have a transformative impact on cognitive modeling. Rigorous tests of cognitive models of attention, memory, learning, and decision-making are often limited to simplistic stimulus spaces because modeling realistic visual inputs is often highly challenging. Using the LMMs to serve as perceptual modules that extract features that a human would extract given the same visual input could enable the scaling up of cognitive models to more realistic settings. Advancements in deep neural networks have already been applied to cognitive science, leading to more general models useful in practical applications, such as decision-making in medicine (Hasan et al., 2022; Holmes et al., 2020; Rahgooy et al., 2022) and economics (Horton, 2023; Korinek, 2023). The present research path of having multimodal models reproduce human perceptual judgments should enhance the application of formal cognitive models to these real-world settings even more.

## Data Availability

Supplemental material, including all the prompts, is available at https://osf.io/za847/. The complete dataset, including rock images and participant ratings, is available at https://osf.io/cvwu9/. None of the reported studies were preregistered.

## Footnotes

1 In brief, rocks with “conchoidal fracture” have scalloped, sea-shell-shaped surfaces that are produced when the rocks are fractured; rocks with “pegmatitic structure” have large banded crystals embedded in a separate groundmass. For illustrations, see Nosofsky, Sanders, Meagher, and Douglas (2020).

2 Despite the instructions for the models to return numerical dimensions to the hundredths place and to use the full array of available values, the outputs most predominantly appeared in increments of 0.05. Given the enormous complexity of the internal workings of the models, we can only speculate about the reason for this outcome. It is possible that because the LMMs are trained on human data, and humans are more likely to respond, say, with 3.05 than 3.04, this rounding bias is present in the LMMs.

## Code availability

The code is available at https://github.com/cogneuroai/multimodal-models-rock.
